# Compact Nonvolatile Reconfigurable Mode Converter by Sb_2_S_3_ Embedded in 4H-Silicon-Carbide-on-Insulator Platform

**DOI:** 10.3390/nano15090689

**Published:** 2025-05-01

**Authors:** Danfeng Zhu, Junbo Chen, Shaobin Qiu, Dingnan Deng, Jinming Luo

**Affiliations:** Meizhou Intelligent Photoelectric Detection Application Engineering Technology Research Center, School of Physics and Electrical Engineering, Jiaying University, Meizhou 514015, China; junbochen1991@163.com (J.C.); qshaobin@gmail.com (S.Q.); deenan@jyu.edu.cn (D.D.); zsuljm@163.com (J.L.)

**Keywords:** nonvolatile switching, reconfigurable mode conversion, phase-change material, Sb_2_S_3_, 4H-SiC

## Abstract

Nonvolatile switching is emerging and shows potential in integrated optics. A compact nonvolatile reconfigurable mode converter implemented on a 4H-silicon-carbide-on-insulator (4H-SiCOI) platform with a footprint of 0.5 × 1 × 1.8 μm^3^ is proposed in this study. The functional region features an Sb_2_S_3_ film embedded in a 4H-SiC strip waveguide. The functionality is achieved through manipulating the phase state of the Sb_2_S_3_. The high refractive index contrast between the crystalline Sb_2_S_3_ and 4H-SiC enables high-efficiency mode conversion within a compact footprint. The incident TM0 mode is converted to the TM1 mode with a high transmittance (*T*) beyond 0.91 and a mode purity (*MP*) over 91.72% across the 1500–1600 nm waveband. Additionally, when the Sb_2_S_3_ transitions to its amorphous state, the diminished refractive index contrast efficiently mitigates the mode conversion effect. In this state, the TM0 mode propagates through the functional region with minimal perturbation, exhibiting *T* ≥ 0.99 and *MP_TM_*_0_ ≥ 97.65% within a 1500–1600 nm waveband. Furthermore, the device performances were investigated under partially crystallized states of Sb_2_S_3_. The proposed structure offers a broad range of transmittance differences (−16.42 dB ≤ Δ*T* ≤ 17.1 dB) and mode purity differences (−90.91% ≤ Δ*MP* ≤ 96.11%) between the TM0 mode and TM1 mode. The proposed device exhibits a high robustness within ±20 nm Δ*l* and ±10 nm Δ*w*. We believe that the proposed multi-level manipulation can facilitate a large communication capacity and that it can be deployed in neuromorphic optical computing.

## 1. Introduction

Phase-change materials (PCMs), a category of compounds, possess amorphous (disordered) and crystalline (ordered) structural states, enabling dramatic changes in their optical properties, e.g., refractive index (*n*) and extinction coefficient (*k*) [1]. The phase states are switched reversibly by external electric or optical stimuli. Furthermore, PCMs stably maintain their phase states without the application of constant power, resulting in huge potential in terms of nonvolatile optical switching and memory applications [2]. Ge_2_Sb_2_Te_5_ (GST), a well-established PCM, provides a high refractive index contrast (Δ*n*~2.8) near the wavelength of 1550 nm but suffers from a large extinction coefficient in both the amorphous and crystalline states [3]. In order to minimize the optical loss, Ge_2_Sb_2_Se_4_Te_1_ (GSST) has been explored, offering a refractive index contrast of ~1.7 and an ultralow loss in the amorphous state [4]. However, the extinction coefficient in the crystalline state should not be ignored. To deal with this issue, Muskens et al. [5] reported two wide-bandgap PCMs, i.e., Sb_2_Se_3_ and Sb_2_S_3_, with ultralow losses (*k* < 10^−5^) in both the amorphous and crystalline states. The low-loss Sb_2_S_3_ features a high refractive index contrast of 0.6 in the telecommunications C-band at 1550 nm, thereby rendering it highly suitable for nonvolatile optical switching in programmable photonic integrated circuits (PICs).

Mode-division multiplexing (MDM) leverages distinct spatial modes of light as independent data channels, and it is acknowledged as a promising technique for solving the exponential growth in data consumption [6]. A critical component of MDM is integrated mode-order converters, which enable seamless transitions between waveguide modes, unlocking the potential of spatial multiplexing in PICs. The principles of mode conversion can be summarized into three types: mode reconstitution, mode coupling, and mode evolution [7]. Mode reconstitution converters rely on the manipulation of the phase relationship within the eigenmodes, employing Mach–Zehnder interferometers (MZIs) [8,9], grating-assisted couplers [10,11], slot waveguides [12,13], and metawaveguides [14,15] dedicated to mode decomposition, phase shift, and mode reconstitution. Mode coupling converters need to fulfill the phase-matching condition between the fundamental and high-order modes through asymmetric directional couplers [16,17], Bragg gratings [18,19], metasurfaces [20,21], and so on. Mode evolution converters are based on the adiabatic transformation of the mode field, which fundamentally requires close matching of the modal effective indices. The reported schemes mainly comprise multichannel branching waveguides [22], asymmetric Y-junctions [23], and tapered waveguides [24,25]. Furthermore, reconfigurable mode-order converters can provide flexibility, scalability, and adaptability, crucial for advancing optical technologies in communication, sensing, quantum systems, and beyond. The ability to dynamically manipulate spatial modes opens new avenues for innovation across interdisciplinary applications. Reconfigurable mode-order converters assisted by PCMs, e.g., GSST [26,27], Sb_2_S_3_ [28], and Sb_2_Se_3_ [29], have been explored and developed. These devices deliver remarkable performance but compromise on their compact device footprint.

Herein, we propose a nonvolatile reconfigurable mode converter implemented on the emerging 4H-SiCOI platform. In the functional region, the Sb_2_S_3_ film is embedded in a 4H-SiC slab waveguide with a compact footprint of 0.5 × 1 × 1.8 μm^3^. When the Sb_2_S_3_ is in its crystalline state, its refractive index at a wavelength of 1550 nm is higher than that of 4H-SiC. Such a high refractive index contrast results in remarkable mode conversion. On the condition that the Sb_2_S_3_ is switched to its amorphous state, this mode-conversion effect becomes negligible due to the low refractive index contrast between the 4H-SiC and the amorphous Sb_2_S_3_ (a-Sb_2_S_3_). Through this, a nonvolatile reconfigurable mode-order converter is achieved that is assisted via manipulating the phase state of the Sb_2_S_3_.

## 2. Design and Principle

**Figure 1:**
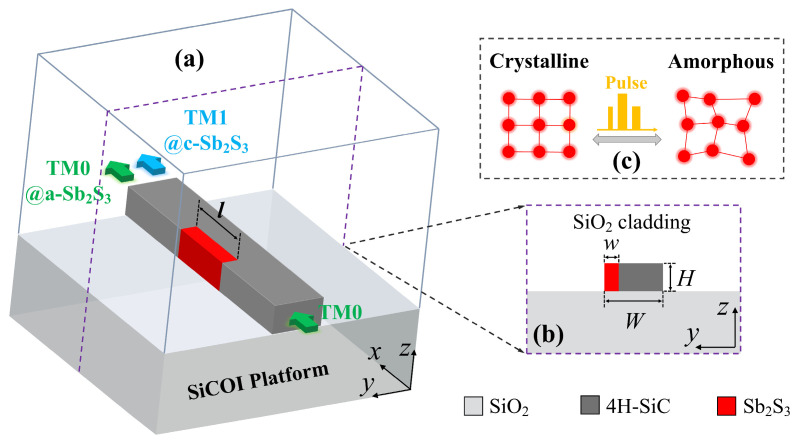
(**a**) Schematic and (**b**) cross-section of the proposed nonvolatile reconfigurable mode converter. (**c**) The typical phase transformation process of Sb_2_S_3_.

A schematic diagram of the method is sketched in Figure 1a,b. The proposed nonvolatile reconfigurable mode converter is deployed on the emerging 4H-SiC platform. In order to perfectly support both the TM0 and TM1 modes, the width *W* and height *H* of the 4H-SiC strip waveguide were set to 1 μm and 0.5 μm, respectively. An Sb_2_S_3_ film, a low-loss PCM in both crystalline and amorphous states near the 1550 nm wavelength, is embedded in a 4H-SiC strip waveguide. To improve the fabrication accuracy and reduce the fabrication difficulty, its height *h* was also fixed at 0.5 μm. Then, its width *w* and length *l* were separately set to 0.22 μm and 1.8 μm through parametric sweeps for attaining the highest mode conversion efficiency. As depicted in Figure 1c, the crystalline and amorphous states of Sb_2_S_3_ can be converted reversibly and fast by electrical or optical stimulations. The refractive index of Sb_2_S_3_ will change with the phase state. According to this rule, a high refractive index contrast exists between the 4H-SiC and the crystalline Sb_2_S_3_ (c-Sb_2_S_3_), which enables intense mode decomposition, phase shifting, and mode reconstitution effects within the specially designed structure, resulting in a remarkable mode conversion between the TM0 mode and TM1 mode. Once the Sb_2_S_3_ is switched in the amorphous state, the refractive index contrast becomes low, resulting in the mode conversion effect being negligible. Thus, a reconfigurable TM0-to-TM1 mode conversion is realized by the manipulation of the Sb_2_S_3_ within a compact footprint of 0.5 × 1 × 1.8 μm^3^.

**Figure 2:**
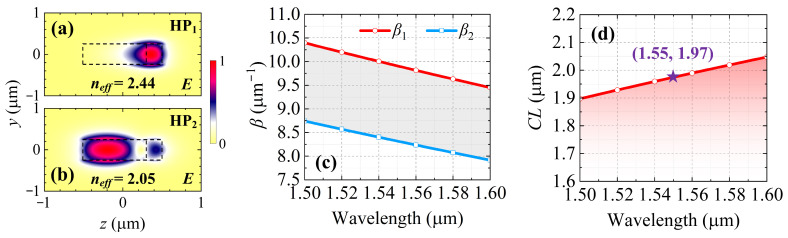
The calculated electric field profiles of (**a**) HP_1_ and (**b**) HP_2_. The areas surrounded by black dotted lines separately refer to the 4H-SiC waveguide and the embedded Sb_2_S_3_ film. (**c**) The propagation constants of HP_1_ and HP_2_ within the functional region. (**d**) The calculated coupling length (*CL*) with a varying working wavelength.

The key element is the mode conversion that happens with the crystalline Sb_2_S_3_. The hybridized-mode interface method is used for the mode conversion [10]. Figure 2a,b depict the two lowest-order hybridized supermodes (HP_1_ and HP_2_), which generate, produce the phase shift, and synthesize in the specially designed functional region. In the process of the mode exchange, the transfer matrixes are described as follows:

(1) $$M=\left[\begin{array}{cc} \kappa_{11} & \kappa_{12}\\ \kappa_{21} & \kappa_{22} \end{array}\right],$$

and

(2) $$L=\left[\begin{array}{cc} e^{-j(\beta_{1}-\beta_{2})l} & 0\\ 0 & 1 \end{array}\right],$$

where *κ_mn_* (*m*, *n* = 1, 2) denotes the coupling coefficient between the incident TM(*n* − 1) mode and the hybridized supermode HP_m_, *β*_1_ and *β*_2_ denote the propagation constants of HP_1_ and HP_2_, respectively, and *l* is the length of the Sb_2_S_3_ film. As the middle functional region is mirror-symmetrical, the transfer matrix *M* can be written as *M^T^* because of the optical reciprocity [30]. The matrix elements of *M* and *M^T^* should fulfill the following: $\kappa_{ik}^{2}+\kappa_{jk}^{2}\leq 1$ and $\kappa_{ki}^{2}+\kappa_{kj}^{2}\leq 1$(*i* ≠ *j* and *k* = 1, 2). According to the transfer matrix method, the output of the converter is expressed as follows:

(3) $$\left[\begin{array}{c} k_{\mathrm{out}1}\\ k_{\mathrm{out}2} \end{array}\right]=M^{T}LM\left[\begin{array}{c} k_{\mathrm{in}1}\\ k_{\mathrm{in}2} \end{array}\right]=\left[\begin{array}{cc} e^{-j(\beta_{1}-\beta_{2})l}\kappa_{11}^{2}+\kappa_{21}^{2} & \kappa_{21}\kappa_{22}+\kappa_{11}\kappa_{12}e^{-j(\beta_{1}-\beta_{2})l}\\ \kappa_{21}\kappa_{22}+\kappa_{11}\kappa_{12}e^{-j(\beta_{1}-\beta_{2})l} & e^{-j(\beta_{1}-\beta_{2})l}\kappa_{12}^{2}+\kappa_{22}^{2} \end{array}\right]\times \left[\begin{array}{c} k_{\mathrm{in}1}\\ k_{\mathrm{in}2} \end{array}\right],$$

where *κ*_in_ *_m_* and *κ*_out_ *_m_* denote the complex amplitudes of the incident TM(*m* − 1) mode and the output TM(*m* − 1) mode (*m* = 1, 2), respectively. To attain a complete mode conversion, it must achieve M^T^LM = [0 1; 1 0], which should satisfy the conditions |*k_mn_*| = sqrt(2)/2 and e^−j(*β*1−*β*2)*l*^ = −1. To ensure the first condition |*k_mn_*| = sqrt(2)/2, various structural designs have been reported, such as Mach–Zehnder interferometers, gratings, slot waveguides, and so on. Herein, we chose a high-refractive-index material (crystalline Sb_2_S_3_) to be inlaid in a slab 4H-SiC waveguide for a compact footprint. Such a specially designed structure can support the pair of desired hybridized supermodes.

Apart from the optimal coupling coefficients, the second condition is e^−j(*β*1−*β*2)*l*^ = −1, i.e., the desired phase-shifting is equally crucial. To calculate the phase shift between HP_1_ and HP_2_, boundary model analyses were conducted by a finite-difference eigenmode (FDE) solver (Ansys Mode Solutions: Waveguide Simulator) [31]. The effective refractive index is expressed as follows [32]:

(4) $$n_{eff}=\beta \frac{\lambda }{2\pi },$$

where *β*, i.e., the propagation constant, is the wavevector along the guiding direction *x*-axis. The effective refractive indices (*n_eff_*) of HP_1_ and HP_2_ were calculated as 2.445 and 2.052 with a wavelength of 1550 nm, respectively. The spectral responses of the propagation constants are depicted in Figure 2c. It can be deduced from the second condition, e^−j(*β*1−*β*2)*l*^ = −1, that the π phase shift is vital for the mode conversion. The corresponding coupling length (*CL*) is described as follows [33]:

(5) $$CL=\frac{\pi }{\Delta \beta }=\frac{\pi }{\left(\beta_{1}-\beta_{2}\right)},$$

which is illustrated in Figure 2d. The sample (1.55, 1.97) means that the π phase shift can be obtained at a wavelength of 1550 nm when the coupling length is equal to 1.97 μm. Therefore, the length *l* of the Sb_2_S_3_ was initially fixed to 1.97 μm, which is further optimized in the three-dimensional simulation for propagation in Section 3.

## 3. Simulation and Performance

**Figure 3:**
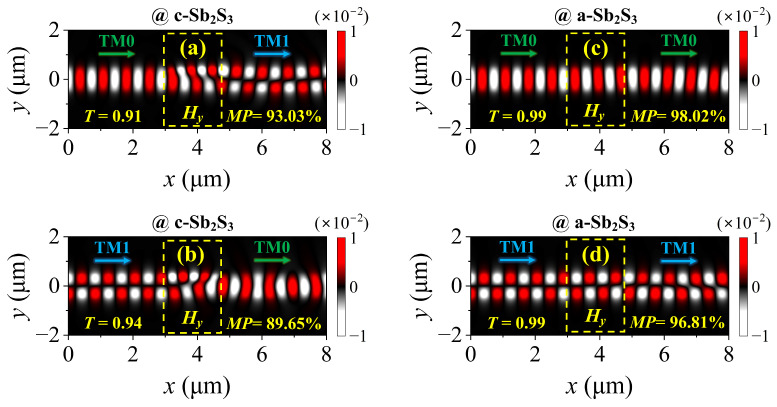
The magnetic field component *H_y_* profiles with the incident (**a**) TM0 mode and (**b**) TM1 mode for crystalline Sb_2_S_3_. The magnetic field component *H_y_* profiles with the incident (**c**) TM0 mode and (**d**) TM1 mode for amorphous Sb_2_S_3_. The rectangles surrounded by yellow dotted lines refer to the functional region.

To calculate the device’s performance, we utilized a three-dimensional finite-difference time-domain (3D FDTD) method (Ansys Lumerical FDTD Solutions: 3D Electromagnetic Simulator) [34]. The refractive indices of silica and 4H-SiC were set to 1.44 and 2.6, respectively. The crystalline and amorphous Sb_2_S_3_ contents were set to 3.308 and 2.716, respectively. The simulated area had dimensions of 5 × 5 × 8 μm^3^. The boundary conditions were set to the perfectly matched layers (PMLs). The mesh-override section was fixed to *dx* = *dy* = *dz* = 10 nm to guarantee precision in the simulation. The transmittances of both the TM0 and TM1 modes were extracted by a mode expansion solver [35]. The target length *l* was optimized to 1.8 μm from 1.97 μm by parameter sweeping to attain the highest mode conversion efficiency. The finite-size field distributions and boundary conditions in the 3D FDTD simulations influenced the phase accumulation, resulting in an optimal length shift. When the inlaid Sb_2_S_3_ was crystalline, the proposed structure converted the incident TM0 mode to the TM1 mode with a high transmittance (*T*) of 0.91 and a mode purity (*MP*) of 93.03% at a wavelength of 1550 nm, as depicted in Figure 3a. The transmission losses were primarily caused by the high refractive index contrast between the crystalline Sb_2_S_3_ and the 4H-SiC, which can be mitigated through introducing some structural elements such as tapers, smoothing of the index transitions, or field matching at the expense of higher fabrication accuracy and difficulty. The mode purity is defined as follows:

(6) $$MP\;(\%)=\frac{T_{TMi}}{T_{total}}\times 100\%,$$

where *T_TMi_* denotes the transmittance of the output TMi mode (*i* = 0, 1), and *T_total_* is the total transmittance. Actually, apart from the output dominant TM1 mode, the other output power consists of the remaining TM0 mode and a higher-order mode (i.e., the TM2 mode). According to the optical reciprocity of the mirror-symmetrical functional region, it can be observed in Figure 3b that the used TM1 mode was converted to the TM0 mode with *T* = 0.94 and *MP* = 89.65%. Such a mode conversion relies on the high refractive index contrast between the 4H-SiC and the crystalline Sb_2_S_3_. However, abrupt refractive index transitions at the material interfaces induce significant light scattering and Fresnel reflections, which impose inherent limitations on the mode conversion efficiency. The proposed approach can obtain a compact device footprint but compromises in terms of the mode conversion efficiency. Once the Sb_2_S_3_ is switched to its amorphous state, its refractive index contrast becomes low, causing the mode conversion effect to disappear. Figure 3c,d show that the TM0 and TM1 modes propagated smoothly through the proposed structure with an ultrahigh transmittance (0.99) and mode purity (98.02% and 96.81%).

**Figure 4:**
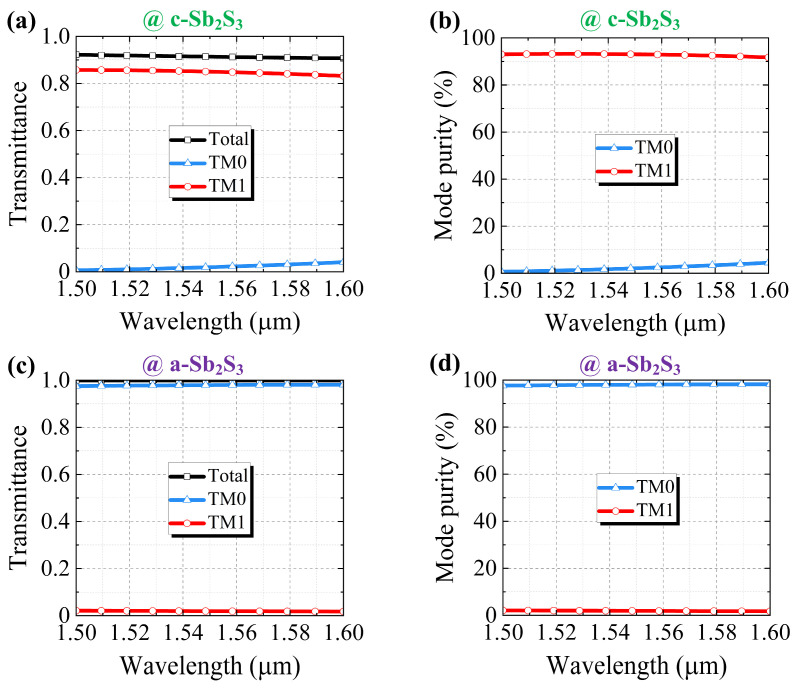
Spectral response of (**a**) transmittance (*T*) and (**b**) mode purity (*MP*) with c-Sb_2_S_3_. Spectral response of (**c**) *T* and (**d**) *MP* with a-Sb_2_S_3_.

The spectral responses of the transmittance and mode purity were calculated with the incident TM0 mode with a 1500–1600 nm waveband. As illustrated in Figure 4a, the transmittance of the TM1 mode (*T_TM_*_1_) remained beyond 0.83, and the transmittance of the TM0 mode (*T_TM_*_0_) remained below 0.041 at a 1600 nm wavelength. As shown in Figure 4b, the mode purity of the TM1 mode (*MP_TM_*_1_) remained above 91.72%, and the mode purity of the TM1 mode (*MP_TM_*_0_) remained lower than 4.49% at a 1600 nm wavelength. Obviously, the TM0-to-TM1 conversion was remarkable with the crystalline Sb_2_S_3_. Figure 4c illustrates that the used TM0 mode was almost unaffected through the proposed structure with *T_TM_*_0_ ≥ 0.97 and *T_TM_*_1_ ≤ 0.021 at a 1500 nm wavelength. It is worth noting, as shown in Figure 4d, that *MP_TM_*_0_ ≥ 97.65% and *MP_TM_*_0_ ≤ 2.1% at a 1500 nm wavelength. It can be inferred that the mode conversion effect became negligible with the amorphous Sb_2_S_3_.

## 4. Discussion of the Partially Crystallized States of Sb_2_S_3_

**Figure 5:**
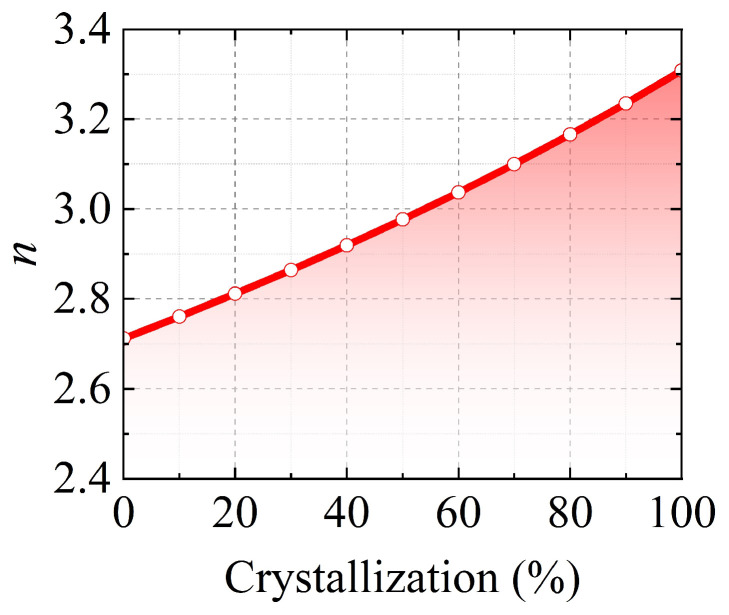
The refractive index *n* of partially crystallized Sb_2_S_3_.

Apart from their crystalline and amorphous states, phase-change materials (PCMs) also possess intermediate states. It is rather challenging to ascertain whether PCMs are in a completely crystalline or amorphous state. It is essential to conduct an investigation into the performances of devices in intermediate states. Multi-level partially crystallized states have been employed in diverse applications, e.g., multi-level memory [36], image displays [37,38], and photonic convolutional neural networks [3]. Sb_2_S_3_ is an emerging PCM that exhibits intriguing intermediate states between its amorphous and crystalline phases. These states are typically induced by normally incident laser pulses and have been validated through Raman spectroscopy. An intermediate state denotes a composite of amorphous and crystalline molecules, characterized by a specific crystallization ratio. The precise selection of peak intensities and durations of control pulses is indispensable for achieving an exact intermediate state. The corresponding effective dielectric constant *ε_eff_* can be estimated via the effective medium theory in combination with the Lorent–Lorenz relation, as follows [39,40]:

(7) $$\frac{\varepsilon_{eff}(\lambda )-1}{\varepsilon_{eff}(\lambda )+2}=m\frac{\varepsilon_{c}(\lambda )-1}{\varepsilon_{c}(\lambda )+2}+(1-m)\frac{\varepsilon_{a}(\lambda )-1}{\varepsilon_{a}(\lambda )+2}$$

where *ε_c_* and *ε_a_* denote the wavelength-dependent permittivity of crystalline and amorphous Sb_2_S_3_, respectively. *m* is the crystallization ratio, ranging from 0 (amorphous) to 1 (crystalline). The calculated refractive indices *n* are shown in Figure 5, with 10% crystallization ratio step. The theoretical refractive indices were used in the simulations to calculate the device performances.

**Figure 6:**
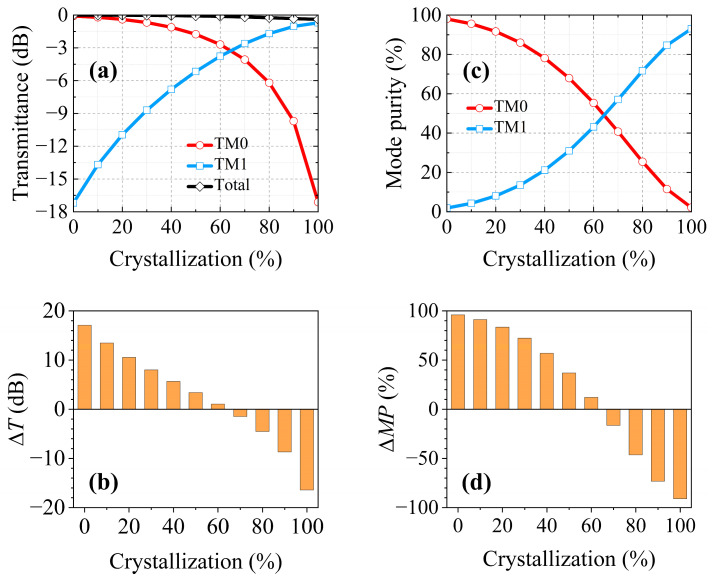
The TM0 mode was incident with a wavelength of 1550 nm. The performances of the partially crystallized GSST, encompassing the (**a**) transmittance, (**b**) transmittance difference Δ*T* between the output TM0 mode and TM1 mode, (**c**) mode purity, and (**d**) mode purity difference Δ*MP* between the output TM0 mode and TM1 mode.

The device performances encompass the transmittance (*T*), transmittance difference between the TM0 mode and TM1 mode (Δ*T*), mode purity (*MP*), and mode purity difference between the TM0 mode and TM1 mode (Δ*MP*). The TM0 mode was used in the proposed structure with a wavelength of 1550 nm. Figure 6 depicts the relationship between the above four performances and the crystallization ratio. It can be observed in Figure 6a that *T_TM_*_0_ diminished as *T_TM_*_1_ escalated with the increase in the crystallization ratio. The reason lies in the fact that the mode conversion effect was enhanced as the crystallization ratio was increased. Figure 6b depicts the transmittance difference between the TM0 mode and TM1 mode (Δ*T = T_TM_*_0_ *− T_TM_*_1_), ranging from −16.42 dB to 17.1 dB. When the crystallization ratio was 63%, the transmittances of the TM0 and TM1 modes were equal, so Δ*T* = 0 dB. As illustrated in Figure 6c, the mode purities of the output TM0 and TM1 modes both equalled 50% at a crystallization ratio of 63%. It is worth noting that the mode purity difference (Δ*MP = MP_TM_*_0_ *− MP_TM_*_1_) possessed a wide range, from −90.91% to 96.11%. To sum up, the proposed structure can provide a broad transmittance difference (−16.42 dB ≤ Δ*T* ≤ 17.1 dB) and mode purity difference (−90.91% ≤ Δ*MP* ≤ 96.11%) between the TM0 mode and TM1 mode by controlling the phase state of the Sb_2_S_3_. Such multi-level manipulation could attain a large communication capacity and could be deployed in convolutional neural networks for neuromorphic optical computing [3].

## 5. Robustness Analysis and Suggested Fabrication

**Figure 7:**
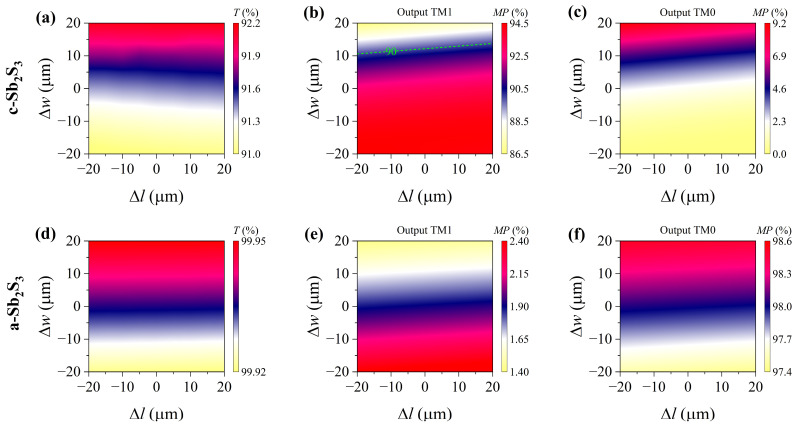
Robustness analysis. (**a**) Transmittance (*T*) and mode purity (*MP*) of output (**b**) TM0 mode and (**c**) TM1 mode with varying Δ*l* and Δ*w* for crystalline Sb_2_S_3_. (**d**) *T* and *MP* of output (**e**) TM0 mode and (**f**) TM1 mode with varying Δ*l* and Δ*w* for amorphous Sb_2_S_3_. The TM0 mode is used with a wavelength of 1550 nm.

**Figure 8:**
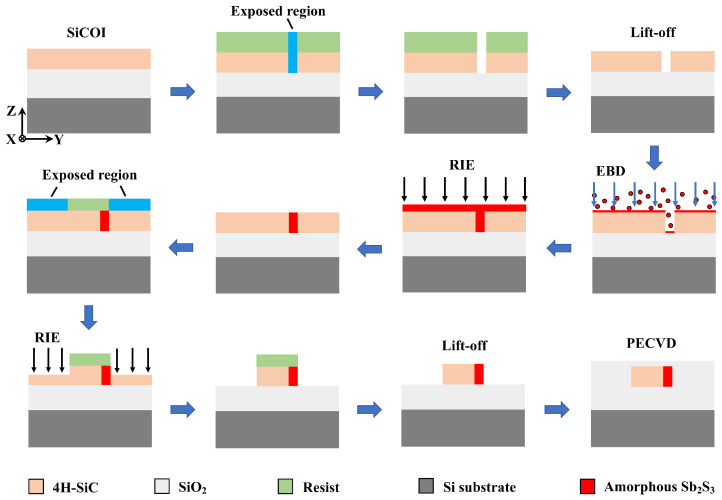
Suggested fabrication method employing two steps of EBL: a resist step followed by RIE and EBD to pattern the amorphous Sb_2_S_3_, and a resist step followed by RIE to define the 4H-SiC waveguide.

As a theoretical study, it was necessary to conduct a robustness analysis. In the functional region, the inlaid Sb_2_S_3_ film is pivotal, so the variations in its length Δ*l* and width Δ*w* were considered. In the simulations, the TM0 mode was used in the proposed structure with a wavelength of 1550 nm. When the Sb_2_S_3_ was in its crystalline state, the incident TM0 mode was converted into the TM1 mode. As depicted in Figure 7a, the transmittance remained above 91% within ±20 nm deviations of Δ*l* and Δ*w*. It should be noted, as shown in Figure 7b, that the mode purity of the output TM1 mode remained beyond 90% within ±20 nm Δ*l* and ±10 nm Δ*w*. Figure 7c illustrates that the mode purity of the remaining output TM0 mode was below 5.28% within ±20 nm Δ*l* and ±10 nm Δ*w*. In a way, the width *w* of the Sb_2_S_3_ is more sensitive than the length *l*. Additionally, when the Sb_2_S_3_ was switched to its amorphous state, the mode conversion effect became inappreciable because of the low refractive index contrast. As shown in Figure 7d, the transmittance remained over 99.92%. It can be observed in Figure 7e,f that *MP_TM_*_1_ ≤ 2.4% and *MP_TM_*_0_ ≥ 97.4% were achieved within ±20 nm deviations of Δ*l* and Δ*w*. In short, the proposed device held its functionality within ±20 nm Δ*l* and ±10 nm Δ*w*. Finally, the suggested fabrication process is sketched in Figure 8. Commercial high-purity semi-insulating 4H-silicon carbide on insulator (4H-SiCOI) material can be fabricated through sublimation growth [41]. An amorphous Sb_2_S_3_ film can be deposited using electron-beam deposition (EBD) [42,43,44,45]. The cladding material, silica (SiO_2_), is deposited onto the 4H-SiC substrate via the process of plasma-enhanced chemical vapor deposition (PECVD). The process for creating the 4H-SiC strip pattern and performing Sb_2_S_3_ lift-off necessitates two-step electron-beam lithography (EBL) procedures, succeeded by reactive ion etching (RIE). The phase state of the Sb_2_S_3_ film can be manipulated through a single-pulse laser with over 7000 switching cycles between amorphous and crystalline Sb_2_S_3_ [46,47]. Intermediate states can be reproducibly achieved through precise control of external laser pulses, and this has been confirmed via Raman spectroscopy. We anticipate that this will be conducive to the fabrication process.

## 6. Conclusions

In conclusion, a compact nonvolatile reconfigurable mode converter is proposed, deployed on a 4H-silicon-carbide-on-insulator (4H-SiCOI) platform with a footprint of 0.5 × 1 × 1.8 μm^3^. The functional region features an Sb_2_S_3_ film inlaid in a 4H-SiC strip waveguide. The functionality is realized via manipulating the phase state of the Sb_2_S_3_. When the Sb_2_S_3_ is crystalline, the high refractive index contrast between the crystalline Sb_2_S_3_ and 4H-SiC facilitates high-efficiency mode conversion. The incident TM0 mode is converted into the TM1 mode with a high transmittance (*T*) of 0.91 and a mode purity (*MP*) of 93.03% at a wavelength of 1550 nm. It is worth noting that *T* ≥ 0.91 and *MP_TM_*_1_ ≥ 91.72% were achieved across a 1500–1600 nm waveband. Once the Sb_2_S_3_ transitions to its amorphous state, its refractive index contrast is significantly diminished, thereby rendering the mode conversion effect negligible. The used TM0 mode was barely affected through the functional region, with a high transmittance of 0.99 and a mode purity of 98.02% at a wavelength of 1550 nm. It should be noted that *T* ≥ 0.99 and *MP_TM_*_1_ ≥ 97.65% were maintained within a 1500–1600 nm waveband. Furthermore, the proposed structure exhibits remarkable versatility, offering a broad range of transmittance differences (−16.42 dB ≤ Δ*T* ≤ 17.1 dB) and mode purity differences (−90.91% ≤ Δ*MP* ≤ 96.11%) between the TM0 mode and TM1 mode via precise control of the Sb_2_S_3_ phase state. The fabrication tolerance analyses revealed its robust performance, maintaining stability within ±20 nm Δ*l* and ±10 nm Δ*w*. We believe this innovative approach to multi-level manipulation not only facilitates enhanced communication capacity but also holds significant potential for deployment in neuromorphic optical computing systems. The unique characteristics and robust performances position it as a promising candidate for advanced photonic applications.

## Data Availability

The raw data supporting the conclusions of this article will be made available by the authors on reasonable request.
